# School factors related to the emotional wellbeing and resettlement outcomes of students from refugee backgrounds: protocol for a systematic review

**DOI:** 10.1186/s13643-019-1016-6

**Published:** 2019-04-30

**Authors:** Deserae Horswood, Jess Baker, Mina Fazel, Susan Rees, Linda Heslop, Derrick Silove

**Affiliations:** 1 University of New South Wales, Kensington, New South Wales Australia; 2 Psychiatry Research and Training Unit, University of New South Wales, L1 Mental Health Centre, Liverpool Hospital, Liverpool, New South Wales 2170 Australia; 3 University of Oxford, Oxford, England; 4 Liverpool Hospital, Liverpool, Australia

**Keywords:** Refugee, School, Mental health, Resettlement, Acculturation, Systematic review

## Abstract

**Background:**

Schools can play a vital role in the resettlement of refugee children and their families. Yet, the body of research examining school environmental factors that support the mental health and acculturation of refugee children is methodologically heterogeneous, investigates numerous and disparate school factors, and is often “hidden” in broader qualitative studies. This limits the capacity to apply the findings in a practical manner.

**Methods:**

Based on PRISMA statement principles, we review the relevant literature to investigate the relationship between school climate and the emotional wellbeing and resettlement outcomes of refugee students.

Six electronic databases will be systematically searched: MEDLINE, PsycINFO, Embase, CINAL, Web of Science, and ERIC, supplemented by a systematic review of the grey literature, relevant international websites, and sequential, site-specific internet searches. Finally, subject area experts will be consulted and backward and forward citation searches of included articles will be completed. Two independent reviewers will screen identified articles against eligibility criteria and extract data for included studies. Quality of included studies will be assessed using the Mixed Methods Appraisal Tool (MMAT) for mixed studies reviews. Data will be synthesised using a convergent qualitative narrative approach.

**Discussion:**

Given the centrality of school in the daily lives of resettled refugee children, it is vital to assess the impact of school climate on the psychosocial wellbeing and resettlement trajectories of this population. This review will identify evidence-based school factors which support good mental health and resettlement outcomes for refugee students and make recommendations for translation of this knowledge into the school environment.

**Systematic review registration:**

PROSPERO CRD42017077570

**Electronic supplementary material:**

The online version of this article (10.1186/s13643-019-1016-6) contains supplementary material, which is available to authorized users.

## Background

Children from refugee backgrounds consistently show elevated levels of psychological morbidity relative to children from non-refugee backgrounds [1–3]. Despite this increased risk of psychopathology, mental health services are significantly under-utilised by children from refugee backgrounds, and those that do attend often engage tenuously [4–6]. Once in high-income countries of resettlement, however, all refugee children must attend school. Schools are one of the first and potentially most influential services that refugee children engage with during their initial resettlement and have a sustained influence throughout childhood and adolescence [7, 8]. The school context plays a pivotal role in the socialisation and acculturation processes in the host country [9, 10]. By acculturation is meant the process of psychological and behavioural change that evolves as a consequence of long-term contact with a dominant culture, including learning new languages, norms, and customs [11–13]. In addition, developing a sense of belonging to the school and having opportunities to contribute to the social fabric of the school and wider community are likely to benefit both the refugee child and other children and families at the school [14]. Given its significance in the daily lives of resettled refugee families, schools may be uniquely placed to support the psychosocial wellbeing, acculturation and resettlement of children.

Schools are often the context for psychological interventions with refugee families [15, 16]. These programs, such as the implementation of trauma-focused cognitive behavioural therapy, predominantly address mental health symptomatology (see [17] for a recent review). Even though these targeted interventions can be effective, not all schools have the resources to implement clinical, time-limited mental health interventions led by psychological professionals.

In addition, there is mounting evidence that the broader resettlement context itself has a profound impact on refugee mental health and wellbeing [18, 19]. Bronfenbrenner’s bioecological model of development (1979) highlights that identity, development, and sense of connection are shaped by an individual’s existence within intersecting and competing social systems. This systems model may be particularly relevant for people from refugee backgrounds who must adjust to new environments across all levels, from the *microsystem* (family, close friends, and peers) to the *macrosystem* (economic, political, and ideological systems of the living environment) [20]. Given the practical impediments to specialist intervention in schools and the likely influence of daily factors, attitudes, and relationships in the school system itself, it is important to investigate the influence of the school climate on the wellbeing of refugee children within a multisystem framework.

School climate is a broad construct, including many factors, and is defined as the institutional “norms, values, and expectations that support people feeling socially, emotionally, and physically safe” [21]. Based on an extensive review of school climate research by Thapa and colleagues, the key attributes of a healthy school climate include that it is a safe place with clear rules, respects diversity, is assertive in preventing and responding to bullying, and fosters healthy relationships between students, teachers, and parents [22]. In high-income countries such as the USA, studies of the general student population have consistently linked a positive school climate to student wellbeing across a range of academic, behavioural, and socio-emotional outcomes; and in early adolescence, a positive school climate is predictive of better psychological health [22]. In refugee samples, research has also provided some evidence of the positive effects of the school climate on refugee students’ mental health and wellbeing [18, 19]. In a study of adolescent Somali refugees living in the USA, a greater sense of school belonging was associated with fewer depressive symptoms and higher self-efficacy even after accounting for exposure to prior adversity [23]. Furthermore, increased support from classmates has been associated with high global self-worth scores in Yugoslavian adolescent refugees in Australia [24], whilst acts of discrimination predicted post-traumatic stress disorder and depressive symptoms [25]. These studies highlight the potency of the resettlement experience, of which the school context is key for many refugee students. However, there is yet to be a critical synthesis of research investigating the impact of school climate on key issues of refugee children’s wellbeing.

Whilst many studies have taken up the question of refugee adaptation to the school context, the body of inquiries are methodologically heterogeneous, investigating numerous and disparate school factors, hence limiting the capacity to draw general inferences or apply the findings in a practical manner. Furthermore, many studies do not differentiate between migrant and refugee populations. Refugee children have a distinct experience from migrant children in many ways, not least in their experiences of trauma and family separations, and the disruptions to their schooling [26]. The cumulative body of research supports these differences in showing that refugee students experience greater difficulties in psychological and socio-cultural adaptation than non-refugee immigrant students [27]. A comprehensive review therefore is needed to determine which school factors relate to positive or negative outcomes in the defined refugee student population, ideally in the inter-related domains of mental health, wellbeing, and acculturation.

By drawing together key learnings of the extant research, this review seeks to identify areas of disjunction between research findings and practice, that is, instances where evidence is not being translated into programs and initiatives demonstrated to promote refugee student mental health and engagement [28]. Surveys indicate that many schools do not use or select evidence-based programs, or they use them with poor fidelity [29]. Given the established importance of the post-migration environment and the significance of school in the daily lives of refugee children, there are compelling reasons to synthesise existing research. This will allow clear directives and actionable recommendations regarding how school climate can support good mental health and resettlement outcomes for refugee children.

## Objectives

The review will aim to systematically identify and evaluate studies that investigate the relationship between school climate and refugee student outcomes. The overarching aim comprises a series of specific research questions:

- Which school factors relate to positive or negative mental health outcomes for refugee students (e.g. clinical indicators of distress or mental disorders)?
- Which school factors relate to positive or negative wellbeing outcomes for refugee students (e.g. nonclinical indicators such as subjective wellbeing)
- Which school factors relate to positive or negative resettlement outcomes for refugee students (e.g. acculturation or social capital)?
- Compared to the general school population, which school factors are of unique relevance to the wellbeing and resettlement of students from refugee backgrounds?

## Methods

This protocol was developed in accordance with the Preferred Reporting Items for Systematic Review and Meta-Analysis Protocols (PRISMA-P) guidelines [30] (see Additional file 1) and is registered on PROSPERO, an international register of systematic reviews [31]. Any changes to the protocol will be recorded on PROSPERO. The review can be described using the PICOS(S) outline (population, intervention, comparator, outcome, study design, and setting).

### Population

The population of interest will comprise school students who are first-generation refugees or asylum seekers resettled in any host country. For the purposes of this review and in line with the 1951 United Nations Convention and 1967 Protocol Relating to the Status of Refugees, a refugee is defined as an individual who was forced to flee their country ‘owing to a well-founded fear of being persecuted’, and whose claim has been verified by the United Nations High Commissioner for Refugees (UNHCR); an asylum seeker is an individual seeking protection as a refugee, but whose claim has not yet been assessed [32]. This review will not include studies of non-refugee immigrants or samples of internally displaced populations. Studies of students from the general school population will be included, provided outcomes are reported separately for students from refugee backgrounds. The mean age for participants in the sample must be 21 years or younger. Whilst this review will not consider studies of higher education students (i.e. university or college), this broader age range is employed to account for delays in grade progression experienced by many refugee students, given language, or other barriers.

### Intervention

This review will be concerned with research that describes or measures any element of the school climate. School climate factors include any item that measures a key factor associated with school experience, for example institutional norms, values, expectations, approaches to diversity, bullying, or relationships between peers or teachers. Specific pedagogical strategies or learning content will not be assessed.

### Comparators

As this study is predominantly focused on aspects of the school climate in general, and not the outcomes of specific interventions, the study need not have a control group for inclusion in the review. If incorporated into the study, comparison groups of refugees, asylum seekers, displaced persons, migrants, native-born, or general student populations will be admissible.

### Outcomes

This review will collect data across two outcome themes. The primary outcomes are the mental health and wellbeing of refugee students as assessed by systematic measure such as self-report, family member, clinician, school staff, or routine data. These outcomes could include, but are not limited to, mental disorders, subjective wellbeing, or psychosocial adjustment. The secondary themes are resettlement outcomes, as assessed by self-report, family member, clinician, school staff, or routine data. These outcomes could include, but are not restricted to, social connectedness, social capital, family relationships, identity, acculturation, educational attainment, or attendance outcomes.

### Study design

This review will be a systematic mixed studies review which integrates quantitative, qualitative, and mixed methods studies, and as such all methodologically sound designs will be eligible for inclusion. Multicomponent studies are acceptable, provided they examine at least one factor of school climate and its relationship to wellbeing or resettlement outcomes for refugee students. As previously stated, this review seeks to consider more broadly the effects of the school milieu, that is, substantive concrete factors in the school context over which schools have an element of control. As such, studies where the primary aim was to evaluate outcomes of an intervention, such as cognitive-behavioural interventions set in the school, will be excluded; synthesis of this work has been completed elsewhere. All relevant studies will be assessed regardless of publication type (e.g. journal article, conference publications), country of study, or publication language; however, only those meeting all inclusion criteria will be included. In line with the Centre for Reviews and Dissemination for systematic reviews [33], all non-English language papers will be identified. In the first instance, Google Translate will be used to determine whether the subject area is aligned with our question, and if so, then we will seek more formal translation. Whilst Google translations may result in errors of content and meaning, the importance of including non-English language papers is evident given the international scope of refugee research.

### Setting

For inclusion, studies must be conducted in a formal school setting in the refugees’ resettlement or transition country, from kindergarten to high school. Studies set in refugee camp schools will be excluded, as a comparison across these two different settings would complicate interpretation of findings. We anticipate that excluding refugee camp settings will result in an analysis of studies predominantly from middle- and high-income countries. However, there are no formal eligibility criteria excluding specific countries of resettlement.

Special education streams targeting refugee children (for example Intensive English Centres) will be included; however, studies that assess adult education or vocational training will be excluded. Although these education routes are viable options for some students, this review aims to compare studies across traditional primary and secondary school settings where the majority of refugee children will first be placed upon resettlement.

### Information sources

Studies will be identified through the following methods:

1. Electronic databases from medicine, science, and education were selected in order to find studies across all relevant disciplines. The following databases will be systematically searched (see the ‘Search Strategy’ section):
  MEDLINE (via Ovid)
  PsycINFO (via Ovid)
  EMBASE (via Ovid)
  Cumulative Index to Nursing and Allied Health Literature (CINAHL) (via EBSCOhost)
  Web of Science Core Collection
  Education Resources Information Centre (ERIC) (via ProQuest)
2. A systematic approach to grey literature searches will be adopted, guided by methods outlined by Godin and colleagues (2015). Firstly, two electronic databases will be searched (TRIP Database and Open Grey) using strategies developed for the electronic databases. Second, specific relevant websites will be hand-searched: UNHCR, World Health Organisation, Save the Children, and the Refugee Studies Centre – Oxford University. Third, we will conduct Google searches for documents published on the internet. Three searches will be conducted, restricting results to sites that end in ‘.gov’, ‘.org’, or ‘.edu’. In line with Godin and colleagues (2015), the first ten pages of each search’s hits (100 results) will be reviewed, using the title and short text underneath. The limit of ten pages will capture the most relevant hits due to Google’s relevance ranking algorithms.
3. Citation searching: backward and forward citation searches will be completed for included studies. Citation lists of included studies will be checked, as will citation lists of papers citing included studies.
4. Expert consultation: key experts in the field of refugee health and education, as identified by the investigators, will be consulted to identify other items for possible inclusion in the systematic review.

### Search strategy

The MEDLINE search strategy was developed through consultation with a research librarian who has expertise in systematic review searching, using an iterative process of preliminary searches, testing search terms and incorporating new search terms as relevant papers are identified. Databases will be searched using date restrictions (1960–2017, or from inception to 2017 for those established later than 1960) and searching titles, abstracts, as well as mapping subject headings specific to each platform. No language or study design limits will be imposed on the search.

Search terms are broad and simple in order to capture all potentially relevant studies. Terms are grouped according to three core concepts: refugee terms (e.g. refugee, asylum seeker, displaced person), school terms (e.g. school, education, student), and child terms (e.g. child, adolescent). Our review outcomes are broad, thus terms specifically relating to outcome factors are not included in the search. This seems almost counterintuitive, but the reasoning is that including less search terms (i.e. no outcome factors) will result in more inclusive search results, in that all studies that relate to refugee children and schools will be returned. This approach was confirmed through extensive piloting of the search term groupings.

Some databases offer limits regarding age groups (for example Medline searches can be limited to 0–18 years). However, the decision was made to narrow by age using word search terms, not limits. This strategy is informed by guidance that databases inconsistently index these age limits and that not all databases include functionality to limit based on age groupings.

Once the MEDLINE strategy was finalised, the strategy was adjusted to the subject headings, syntax, and operating systems of the other databases. See Additional file 2 for the master search strategy for MEDLINE.

### Data management

Literature search results will be collated in reference management software Endnote X8 and duplicate citations will be removed electronically.

### Selection process

Once search results are collated, two researchers will independently screen the titles and abstracts to determine whether a study meets the general inclusion criteria. Each article will be rated as include, exclude, or unclear. The full text of all articles classified as include or unclear will be retrieved for formal review. Next, two reviewers will independently assess the full text of each study according to the predetermined inclusion criteria. If necessary, researchers will seek additional information from study authors to resolve any concerns about eligibility. Disagreements will be resolved by discussion between the two reviewers or (when unable to be resolved) third author adjudication. Reasons for excluding studies will be recorded. Review authors will not be blind to the journal titles, nor authorship information of the studies.

### Data extraction

Study data will be extracted using standard forms developed based on the Cochrane Effective Practice and Organisation of Care Review Group. Data items to be extracted include study design, study population (e.g. gender, age, country of origin, host country, duration in country, how identified for the study), number of participants, age of participants, gender, school climate factor assessed, outcome measures/informants, language spoken by participants, language study conducted in, comparison group, study site (e.g. primary/secondary school, Intensive English Centre), and study findings. Extraction will be conducted by two researchers. Data extraction forms will first be piloted and amended as necessary. Then, each reviewer will perform extraction on half the included studies, then will review the data extracted by the other reviewer on the second half of the articles. As such, each will complete either initial data extraction or review of data extraction on all included studies.

### Quality of individual studies

Two researchers will independently assess the methodological quality of included studies using the Mixed Methods Appraisal Tool (MMAT) for mixed studies reviews [34]. This tool appraises three methodological domains: mixed method, qualitative, and quantitative (further divided into sub-domains: randomised controlled, non-randomised, and descriptive). Each study will be appraised using the relevant domain criteria, and these scores will then be converted to percentages for comparison across domains, where a low percentage indicates low methodological quality and 100% indicates high quality in that all criteria were met. Disagreements will be resolved through discussion between the two reviewers or, when unable to be resolved, third author adjudication.

### Synthesis

Due to the anticipated methodological heterogeneity of research included in this mixed studies review, quantitative synthesis or meta-analyses will not be possible. In order to appropriately compare the diversity of findings, a convergent qualitative synthesis will be performed [35]. Results from studies that include quantitative, qualitative, and mixed method design will be transformed into qualitative findings such as themes and concepts, and a narrative approach will be used to synthesise these results in relation to the outcomes of interest.

The narrative synthesis will explore the findings within and between each included study as they pertain to the mental health, emotional wellbeing, and resettlement outcomes for participants. An overall assessment of the robustness of the evidence will be ascertained using weightings from the quality appraisals; the strength of evidence for each main outcome variable will be synthesised and presented as key recommendations for policy and practice and to inform future inquiry.

## Discussion

Schools may be uniquely placed to support the acculturation and psychosocial needs of students from refugee backgrounds. In general school populations, school climate factors are linked with positive wellbeing and health indicators [36], yet the implications of these factors for refugee students are not clear. This study will determine the content and quality of research that establishes links between the school climate and mental health, wellbeing, and resettlement outcomes for refugee students. This will move toward closing the wide gap between established research and practice in school-based wellbeing initiatives [28]. By understanding which elements of the school climate are influential to refugee student mental health and resettlement, education departments and schools can effectively channel their limited time and resources to appropriately support this vulnerable population.

## Additional files

Additional file 1: Prisma-P Checklist. Completed Prisma-P checklist with line numbers (PDF 121 kb)

Additional file 2: Search Strategy: Medline – OVID. Exact search terms and groupings of terms to be used for Medline (OVID) (PDF 32 kb)

## Abbreviations

CINAHL: Cumulative Index to Nursing and Allied Health Literature
ERIC: Education Resources Information Centre
PTSD: Post-traumatic stress disorder
UNHCR: United Nations High Commissioner for Refugees
