# Supplementary material for: School factors related to the emotional wellbeing and resettlement outcomes of students from refugee backgrounds: protocol for a systematic review
Source: Syst Rev. 2019 Apr 30;8:107. doi: 10.1186/s13643-019-1016-6 (PMC6492402; doi:10.1186/s13643-019-1016-6)
Supplement: Supplementary file 2 — Search Strategy: Medline – OVID. Exact search terms and groupings of terms to be used for Medline (OVID) (PDF 32 kb) [file 13643_2019_1016_MOESM2_ESM.pdf]

Additional File 2 - Search Strategy: Medline – OVID

1.	refugees/
2.	undocumented immigrants/
3.	refugee*.ti,ab.
4.	(asylum adj3 seek*).ti,ab.
5.	forced migration*.ti,ab.
6.	(displaced adj3 (adolescen* or child* or juvenile* or minor or person* or people* or teen* or young or youth*)).ti,ab.
7.	1 or 2 or 3 or 4 or 5 or 6
8.	schools/
9.	education/ or education, special/
10.	students/
11.	curriculum/ or teaching/
12.	(classroom or class room or curricul* or peer* or school*).ti,ab.
13.	((classroom or class room or education* or learn* or school*) adj3 (based or environment* or multicultur* or program*)).ti,ab.
14.	8 or 9 or 10 or 11 or 12 or 13
15.	exp child/ or adolescent/
16.	(adolescen* or child* or juvenile* or minor or teen* or young or youth*).ti,ab.
17.	15 or 16
18.	7 and 14 and 17
19.	Limit 18 to yr= 1960 -Current
